# A Novel Measurement of Altered Achilles Subtendon Load Sharing 6–12 Months Following Rupture

**DOI:** 10.1002/jor.70182

**Published:** 2026-03-26

**Authors:** Kathryn S. Strand, Todd J. Hullfish, Maggie M. Wagner, Devyn Russo, Max Zawel, Douglas E. Schaubel, Casey Jo Humbyrd, Josh R. Baxter

**Affiliations:** ^1^ Department of Orthopaedic Surgery University of Pennsylvania Philadelphia Pennsylvania USA; ^2^ Department of Biostatistics, Epidemiology and Informatics University of Pennsylvania Philadelphia Pennsylvania USA

**Keywords:** Achilles tendon, rupture, subtendons, ultrasound

## Abstract

Achilles tendon ruptures cause muscle–tendon structural and functional deficits that persist years after the initial injury. A healthy Achilles tendon contains three semi‐independent subtendons that slide relative to each other during muscle contractions in healthy adults. However, such sliding decreases postinjury as load sharing—likely caused by intratendinous adhesions—increases between adjacent subtendons. This study quantifies changes in subtendon load sharing 6–12 months following Achilles tendon rupture when patients are cleared by their surgeon to fully return to physical activities. We combined transverse plane ultrasound imaging with neuromuscular electrical stimulation of individual triceps surae muscles and applied a Kanade–Lucas–Tomasi point tracking algorithm to characterize subtendon behavior. We developed a surrogate measure of subtendon load sharing by quantifying differences in point displacement trajectory angles between select regions within the tendon cross section. In patients recovering from rupture injuries (*n* = 19), subtendon load sharing significantly increased in the injured tendon compared to the contralateral uninjured side during lateral gastrocnemius (*p* = 0.0094), medial gastrocnemius (*p* = 0.021), and soleus stimulations (*p* = 0.048). These differences were not present between right and left legs in the uninjured cohort (*n* = 17). Linear regression analysis also revealed that the presence of tendon injury was significantly associated with subtendon load sharing, with injured tendons showing up to a 44% decrease in subtendon independence compared to the contralateral uninjured tendon during gastrocnemius stimulations.

**Statement of Clinical Significance:** These results propose a novel biomarker of tendon health and suggest muscle‐dependent changes in subtendon function following Achilles tendon rupture.

## Graphical abstract

This study quantifies altered Achilles subtendon function following rupture injury. We developed a surrogate measure of subtendon load sharing by quantifying differences in tissue displacement patterns within the tendon cross section during isolated triceps surae contractions. In patients recovering from rupture injuries (*n* = 19), subtendon load sharing significantly increased in the injured tendon compared to the uninjured side. These differences were not present in healthy young adult controls. These results present a novel biomarker of Achilles subtendon health postinjury.

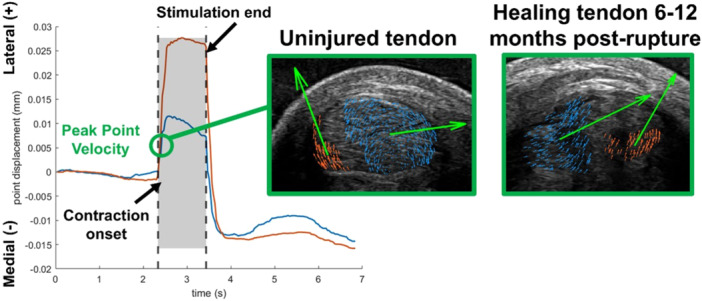

## 1 Introduction

Achilles tendon rupture affects 2.5–47 per 100,000 individuals each year, comprising 10.7% of all tendon and ligament ruptures [1]. Ruptures cause functional deficits including gait abnormalities, altered ankle range of motion, and plantar flexor weakness which persist over 1 year after injury [2]. These deficits are hypothesized to result from alterations in the intrinsic structure and function of the Achilles tendon postinjury. Structurally, the Achilles tendon is comprised of three semi‐independent subtendons, each arising from a head of the triceps surae muscles: the medial gastrocnemius (GM), lateral gastrocnemius (GL), and soleus (SOL). In a healthy Achilles tendon, these subtendons slide relative to each other during active and passive ankle rotations [3, 4], but that sliding is reduced following tendon rupture and repair [5, 6, 7]. This reduction in sliding may result from decreased collagen organization postinjury as well as the formation of fibrotic scar tissue [8]. Moreover, as proper mechanical stimulation during the remodeling phase of tendon healing helps to restore a tendon's native fiber alignment [9], insufficient rehabilitation loading during injury recovery may exacerbate these changes. Standard clinical care following rupture injury consists of immobilization of the ankle for approximately 2–4 weeks immediately following rupture, a gradual return to full weight‐bearing by approximately 3 months, and then a progressive increase in activities such as walking, heel raises, and running [10, 11]. However, clinicians currently lack unified metrics of tendon healing status to signify when a patient is ready to progress to the next stage of rehabilitation. As such, there is a need for more quantifiable biomarkers of tendon health to guide injury rehabilitation, and these biomarkers may lie in altered subtendon function post‐rupture.

The heterogeneous displacement between adjacent Achilles subtendons highlights the unique functional morphology of the individual triceps surae muscles [12]. Prior work demonstrated the effects of altered muscle dynamics and Achilles subtendon sliding in both computational and preclinical models. For example, Handsfield and colleagues demonstrated that lower force differentials between the triceps surae muscles decrease heterogeneous Achilles tendon displacement by 85% [13]. Maas and colleagues also found that selective stimulation of triceps surae muscles in a rat produced differential relative strains between the SOL and GL subtendons depending on knee and ankle joint angles [14]. Together, these results provide evidence for a “muscle‐down” effect in which triceps surae dynamics alter Achilles tendon loading. However, there is also a possibility of a “tendon‐up” effect in which muscle dynamics and their relative contributions to joint load are altered by changes in their common tendon. For example, Franz and Thelen postulate that reduced subtendon sliding in elderly adults unfavorably couples the soleus and gastrocnemii muscles leading to an observed 0.2 Nm/kg reduction in peak ankle moment during walking [15]. Predictive models have shown that increased interfascicular adhesions within the Achilles tendon produce 6% faster peak gastrocnemius fiber shortening velocities and 3% slower soleus shortening velocities [15]. The potential upward effects of altered subtendon behavior on muscle–tendon function, particularly following Achilles tendon rupture when the native tendon structure is highly compromised, highlight the need for quantifiable measures of intratendinous load sharing.

Altered subtendon function and load sharing may be an early indicator of tendon healing status given the reductions in heterogeneous subtendon displacement following Achilles tendon rupture [6, 16, 17]. Achilles tendon healing also benefits from early mechanical stimulation. Prior studies in rats have revealed that mechanical stimulation in the early inflammatory phase of healing improved tendon strength [18]. Studies in humans also revealed improved Achilles tendon elastic moduli with early tensional loading starting 2 weeks post‐rupture [19]. Despite the benefits of early mechanical stimulation, clinicians still lack quantifiable measures of tendon healing status, particularly in early stages of tendon healing, to signify that a patient may progress in their rehabilitation and safely increase tendon loading. Prior studies primarily examine tendon mechanical properties starting 3 months post‐rupture, reporting decreased tendon stiffness around 6 months postinjury [20] and increased longitudinal tendon stiffness 2–6 years postinjury [21]. However, these measurements involve high loading conditions such as walking, heel raises, or maximum voluntary plantar flexor contractions. Due to these high loads, such methods are not suitable to evaluate the tendon in earlier healing stages post‐rupture such as when a patient is still wearing an immobilizing boot to minimize tendon loads and prevent re‐rupture. As such, there is a need for new methods to evaluate Achilles tendon properties at earlier timepoints under low‐load conditions. Moreover, scar formation and adhesions to surrounding tissues post‐rupture [8] may also alter transverse tendon stiffness and lateral load sharing between subtendons. Such properties have not been characterized in the early healing stages following Achilles tendon rupture.

The purpose of this study was to quantify differences in subtendon function between injured Achilles tendons 6–12 months following rupture and uninjured tendons under low‐load conditions using isolated triceps surae muscle stimulations. We hypothesized that the cross section of an injured tendon post‐rupture would display more homogeneous behavior in response to isolated muscle stimulations compared to an uninjured tendon. We also hypothesized that the presence of a tendon injury would be associated with the degree of load sharing within the tendon cross section. The results of this study will present new potential biomarkers of Achilles tendon health postinjury.

## 2 Methods

### 2.1 Study Population

We recruited 19 adults with unilateral Achilles tendon rupture (16 M, 3 F, age: 38 ± 10 years, BMI: 27.8 ± 4.4) who provided informed, written consent to participate in this case‐control study approved by the University of Pennsylvania Institutional Review Board (Protocol 850302). Patients were evaluated 38 ± 13 weeks following surgical intervention (or first clinical visit if treated nonoperatively). We chose to evaluate patients at this stage of healing because they were functionally cleared by their physician to return to sport and physical activity. Prior work has established that subtendon function remains altered up to 1 year postinjury [6, 16, 17]. We evaluated this cohort to determine if our low‐loading protocol would identify similar functional changes within the tendon and thus could later be applied to patients in earlier healing phases. A total of 18 patients had critical zone ruptures, and 1 patient had a rupture at the calcaneal insertion. In total, 14 patients were treated surgically, and the remaining 5 patients were treated nonoperatively. The surgically repaired patients were treated by four different fellowship trained foot and ankle surgeons. These providers used different surgical approaches: 6 patients were treated using a percutaneous repair, 3 patients were repaired using a mini‐open approach that visualized the tendon through a small longitudinal surgical incision over the rupture location, and 5 patients were repaired using an open approach where both the tendon and the repair are fully visualized. We chose not to stratify patients based on their surgical treatment approach because prior clinical studies have failed to identify clear biomechanical differences between initial treatment other than soft tissue healing complications [22, 23, 24]. We also recruited a group of 17 healthy adults (8 M, 9 F, age: 27 ± 6, BMI: 24.3 ± 4.6) with no history of Achilles tendon injuries to compare bilateral differences in subtendon behavior in individuals both with and without tendon injuries. We did not match for age, sex, or activity level between these two study groups because we used our uninjured group specifically to establish side‐to‐side variability in our primary outcome measurement. These sample sizes were determined based on a power analysis described in our previous work [25]. This analysis indicated that a sample size of at least 15 subjects was required to achieve a power of 0.93 (effect size: 0.96, *α* = 0.05) calculated from a pilot study of 8 subjects based on the hypothesis that there would be no differences in the locations of subtendon responses between right and left legs within the tendon cross section.

### 2.2 Ultrasound Imaging and Neuromuscular Electrical Stimulation Protocol

**Figure 1:**
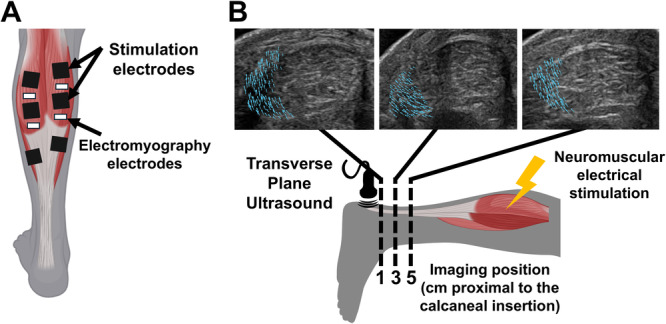
(A) Electromyography and neuromuscular electrical stimulation electrode placement. (B) Imaging protocol and example output, with representative vector fields reflecting localized displacements within the tendon cross section at three imaging locations along the free tendon.

We combined transverse‐plane ultrasound imaging and neuromuscular electrical stimulation of individual triceps surae muscles to quantify localized tendon displacements caused by single muscle contractions similar to previous work [25, 26, 27]. Participants laid prone on a treatment plinth with their knees fully extended and the ankle in a relaxed position hanging freely off the edge of the bed. We fixed a 21 MHz linear ultrasound transducer (L6‐24, LOGIQ E10, GE HealthCare, Chicago, IL, United States) perpendicular to the free tendon 1 cm proximal to the calcaneal insertion point with a custom 3D‐printed fixture to acquire ultrasound images in the transverse plane. We marked the calcaneal insertion point on the skin to guide ultrasound probe placement. We selected ultrasound imaging parameters to optimize image quality: gain: 45, dynamic range: 63, depth: 2–3 cm. We delivered monophasic pulse trains (100 Hz, 400 µs pulse width, 1 s duration) via hydrogel electrode pairs (25 × 38 mm) placed over the largest portion of the muscle bellies of the GM, GL, and SOL muscles [28] (Figure 1A). Following our established methods [25], we increased the stimulation amplitude stepwise by 1–2 mA until there was localized movement of the Achilles tendon visible via ultrasound video but no plantar flexion of the foot, ensuring isolated recruitment of a single triceps surae muscle. To maintain safe loading conditions for healing tendons, it is necessary to keep the loading below the mechanical strength of a suture repair. Our previous experiments in 8 healthy adult subjects demonstrate that this protocol delivers a maximum load of approximately 14.8 N to the tendon [25] which is 95% less than the mechanical strength of sutures used in surgical rupture repair [29], thus maintaining safe, low loading conditions. We delivered the chosen stimulation amplitude to each muscle with the ultrasound probe placed 1, 3, and 5 cm proximal to the calcaneal insertion (Figure 1B), ensuring that the probe remained distal to the soleus muscle–tendon junction. We selected these imaging locations to ensure visualization of the entire free tendon both proximal and distal to the repair site. We also collected electromyography data of the GL and GM to confirm minimal co‐activation of nonstimulated muscles. We only collected electromyography data from the GL and GM muscles during this protocol because our prior work observed minimal co‐activation of the SOL muscle during gastrocnemius activation [25]. We placed hydrogel electromyography recording electrodes (10 × 10 mm) in a bipolar configuration with one electrode positioned between the stimulation pads and the other positioned below the distal pad (Figure 1A). Grounding electrodes were placed over the medial and lateral malleoli. We recorded electromyography activity at 7.5 kHz (Ripple Neuromed, Millcreek, UT, United States) and processed the data using a 20–450 Hz bandpass filter. We calculated the root mean square (RMS) of the signal using a 200 ms moving window with 50 ms displacement. We then calculated the peak GL and GM RMS values during each muscle stimulation and found minimal co‐activations of nonstimulated muscles, with mean peak RMS values < 3.07 mV (Table 1). Our previous work employed similar methods of isolated gastrocnemius stimulations and found peak RMS values of nonstimulated triceps surae muscles averaging < 2.9 mV [25]. Together, these consistently low residual activation levels and absence of observed plantar flexion demonstrate that this method reliably recruits individual triceps surae muscles.

**Table 1:** Mean (± standard deviation) of peak RMS values recorded during individual muscle stimulations in the injured and uninjured cohorts.

	Injured cohort		Uninjured cohort	
	GM peak RMS (mV)	GL peak RMS (mV)	GM peak RMS (mV)	GL peak RMS (mV)
GL stimulations	2.05 ± 1.65	—	2.47 ± 2.56	—
GM stimulations	—	3.08 ± 7.45	—	2.66 ± 3.00
SOL stimulations	1.98 ± 2.21	1.65 ± 1.51	1.47 ± 1.33	1.23 ± 0.82

### 2.3 Data Processing Workflow

**Figure 2:**
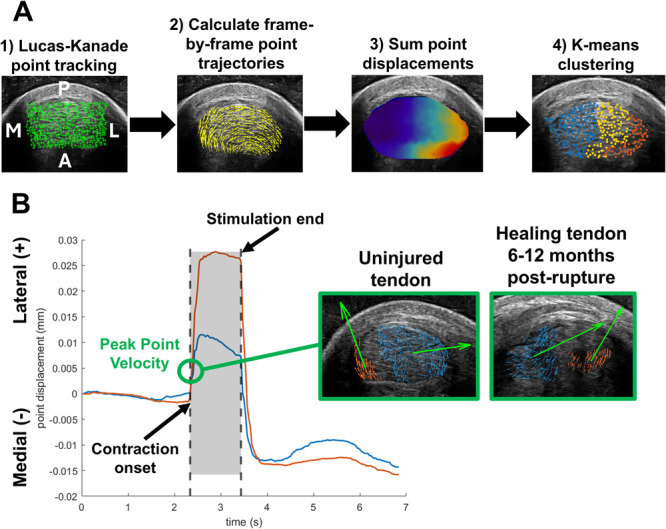
(A) Data processing workflow. (B) Representation of angular difference value calculated as the difference between the mean angle of the displacement vectors between select clusters at the time of peak point velocity immediately following stimulation onset. The left ultrasound image demonstrates a larger angular difference occurring in an uninjured tendon, and the right ultrasound image demonstrates a smaller angular difference occurring in an injured tendon.

Following our previously described methods [25], we used a Kanade–Lucas–Tomasi point tracking algorithm [30, 31] to quantify localized displacements within the tendon cross section during each muscle stimulation. This processing workflow calculated the frame‐by‐frame displacement of 900 corner point eigenfeatures within the tendon cross section. Next, we applied k‐means clustering (*k* = 3 clusters) to group points based on their cumulative displacement during the 1 s stimulation (Figure 2A). The cluster with the lowest cumulative displacement represented nonstimulated tissue, and the cluster with the highest cumulative displacement represented the subtendon of interest, or the region most responsive to the stimulation. The final cluster represented “intermediate tissue” influenced by inter‐subtendon load sharing. We further refined clusters using density‐based clustering (minimum points: 20, epsilon: 35) to isolate cohesive regions for further analysis [25]. Our prior work established that this unsupervised clustering approach was robust and reliably identified tissue regions based on these displacement values, with coefficients of variation < 2% [25]. The displacement of any point at each frame is represented as a vector, so we calculated the mean vector angle of the points within each cluster at the time of peak point velocity immediately following stimulation onset (Figure 2B). Based on prior reports of reduced independent subtendon sliding following rupture [6], we expected the behavior of points to differ more between clusters on the uninjured leg than on the injured leg. To quantify this differential behavior, we calculated the difference in the mean displacement angle between the clusters experiencing the least and greatest cumulative displacement (Figure 2B). We refer to this outcome measure as *angular difference*. A greater angular difference indicates a higher degree of subtendon independence and less homogenous behavior within the tendon cross section, while a lower value indicates increased load sharing between adjacent subtendons and more homogeneous behavior within the tendon.

### 2.4 Statistical Analysis

To evaluate the bilateral differences in subtendon responses within the uninjured and injured cohorts, we performed a two‐tailed Wilcoxon signed‐rank test to compare the angular difference values calculated for each leg. We also performed multiple linear regression analysis to evaluate the correlations of six independent variables with the angular difference outcome in the injured cohort. The independent variables included in the model were age, BMI, sex (M/F), repair type (operative/nonoperative), injury status (injured/uninjured leg), and imaging position along the free tendon (1, 3, and 5 cm proximal to the calcaneal insertion). As the angular difference values were left‐skewed, we transformed the data by taking the natural logarithm prior to running the regression model. We evaluated three regression models, one for each muscle stimulation. All significance levels were set to *p* < 0.05. Statistical analyses were conducted in Python and R. We identified outliers based on the performance of the point tracker. The tracker fails to converge when frame‐to‐frame motion is excessively large, causing it to calculate cumulative displacement values greater than approximately 7 mm (Figure S1). As such values are not physiologically possible given the size of the tendon cross section, we removed trials with calculated displacements greater than this threshold value. We removed 13 data points from the final analysis across both the injured and uninjured cohorts representing < 2.5% of all data. During linear regression analysis, we identified outliers based on Cook's Distance method and removed points with a Cook's Distance value > 4/*n*, where *n* is the number of observations [32].

## 3 Results

**Figure 3:**
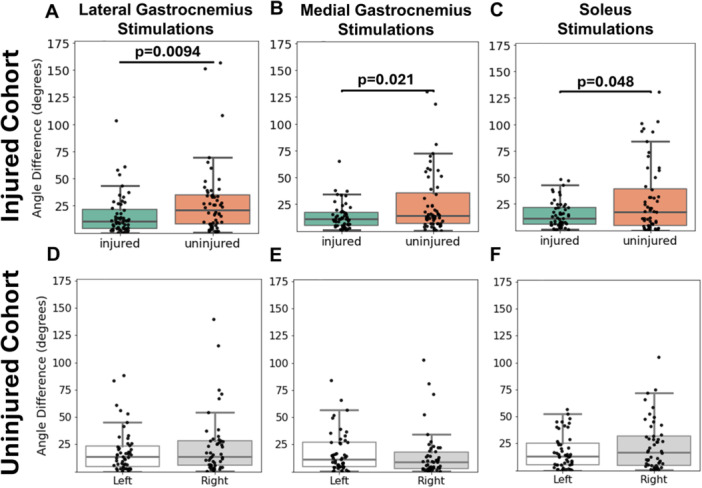
Angular difference values calculated for the injured and contralateral uninjured tendon during (A) GL, (B) GM, and (C) SOL stimulations in the injured cohort. Angular difference values calculated for the right and left tendons in the uninjured cohort during (D) GL, (E) GM, and (F) SOL stimulations. Plots contain data points from all three imaging positions.

Injured tendons displayed more homogenous subtendon behavior in response to individual muscle stimulations compared to the contralateral, uninjured tendon, quantified by a lower angular difference (Figure 3A–C). The mean angular difference on the uninjured side was significantly greater than the injured side during GL stimulations (*p* = 0.0094, effect size: 0.44) (Figure 3A), during GM stimulations (*p* = 0.021, effect size: 0.47) (Figure 3B), and during SOL stimulations (*p* = 0.048, effect size 0.48) (Figure 3C). Angular difference values did not differ between right and left legs in the uninjured cohort during any muscle stimulation (Figure 3D–F). Images of the displacement vector fields from a subset of trials used in these calculations are shown in Figures S2 (injured cohort) and S3 (uninjured cohort).

Linear regression analysis revealed that presence of a tendon injury was significantly correlated with angular difference during GL and GM stimulations. During GL stimulations, injured tendons decreased the angular difference by a factor of 0.563 (43.7% decrease, *p* = 0.0047) (Table 2). During GM stimulations, injured tendons decreased the angular difference by a factor of 0.622 (37.8% decrease, *p* = 0.014) (Table 3). During GL stimulations only, age was also significantly correlated with angular difference, resulting in a 2.64% increase in this outcome measure (*p* = 0.025) (Table 2). There were no significant associations among these variables during SOL stimulations (Table 4).

**Table 2:** Linear regression output for model including GL stimulation data only.

GL stimulations	Estimate (*β*)	Std. error	*p*	Exp (*β*)
(Intercept)	1.939	1.137	0.092	6.952
Sex (F vs. M)	0.097	0.278	0.729	1.101
Treatment (non‐Op vs. Op)	−0.120	0.279	0.669	0.887
3 cm imaging position (vs. 1 cm position)	0.104	0.239	0.663	1.110
5 cm imaging position (vs. 1 cm position)	−0.391	0.242	0.109	0.676
Injury status (inj vs. uninj)	−0.574	0.198	0.0047**	0.563
Age	0.025	0.011	0.025*	1.026
BMI	< 0.0001	< 0.0001	0.954	1.000

*Note:* Asterisk indicates a significant correlation.

* *p* < 0.05;

** *p* < 0.01.

**Table 3:** Linear regression output for model including GM stimulation data only.

GM stimulations	Estimate (*β*)	Std. error	*p*	Exp (*β*)
(Intercept)	3.014	1.067	0.006**	20.369
Sex (F vs. M)	−0.197	0.261	0.452	0.821
Treatment (non‐Op vs. Op)	−0.197	0.254	0.440	0.821
3 cm imaging position (vs. 1 cm position)	−0.274	0.231	0.239	0.761
5 cm imaging position (vs. 1 cm position)	−0.338	0.236	0.156	0.713
Injury status (inj vs. uninj)	−0.475	0.189	0.014*	0.622
Age	0.005	0.010	0.621	1.005
BMI	< 0.0001	< 0.0001	0.477	1.000

*Note:* Asterisk indicates a significant correlation.

* *p* < 0.05;

** *p* < 0.01.

**Table 4:** Linear regression output for model including SOL stimulation data only.

SOL stimulations	Estimate (*β*)	Std. error	*p*	Exp (*β*)
(Intercept)	0.262	1.367	0.848	1.299
Sex (F vs. M)	−0.175	0.343	0.611	0.840
Treatment (non‐Op vs. Op)	0.227	0.326	0.487	1.255
3 cm imaging position (vs. 1 cm position)	−0.231	0.299	0.442	0.794
5 cm imaging position (vs. 1 cm position)	0.024	0.290	0.934	1.025
Injury status (inj vs. uninj)	−0.258	0.240	0.284	0.772
Age	0.026	0.014	0.062	1.026
BMI	0.045	0.031	0.151	1.046

## 4 Discussion

The purpose of this study was to evaluate changes in Achilles subtendon function at 6–12 months post‐rupture under low loading conditions. We developed a surrogate measure of subtendon load sharing termed *angular difference* by quantifying point displacement patterns within localized regions of the tendon cross section. Our results demonstrated that Achilles tendon rupture altered subtendon function in the injured compared to the uninjured legs, and these differences were not present between right and left legs in our healthy adult cohort. Moreover, linear regression analysis revealed that tendon injury status was associated with the relative heterogeneity of subtendon behavior, quantified by the angular difference outcome measure within the Achilles tendon cross section during gastrocnemius stimulations. While other studies have applied simultaneous single‐muscle neuromuscular electrical stimulation with transverse plane ultrasound imaging to understand subtendon morphology [25, 26, 27], this study is the first to apply this method to elucidate differential subtendon function between injured and uninjured tendons. Overall, these results suggest that differences in displacement patterns between localized regions of the tendon cross section may be a new biomarker of tendon health.

This study presents a novel measure of subtendon function that we interpret as the degree of load sharing between adjacent subtendons. In a healthy Achilles tendon, an increased angular difference value indicates that the region corresponding to the stimulated subtendon responds more independently compared to its surrounding tissue. In contrast, in an injured tendon with highly disorganized fibers [8], the stimulated subtendon has more attachments to its neighboring tissue. These intratendinous adhesions cause a subtendon to “pull” on its neighboring tissue when stimulated and produce more homogenous movement within the tendon cross section. Our results illustrate this phenomenon, with angular differences during triceps surae stimulations being significantly lower on injured compared to uninjured legs. The results are further supported by our linear regression models. The coefficients from our regression analyses (Tables 2 and 3) demonstrate that injured tendons have decreased angular difference measurements by approximately 44% during GL stimulations and 38% during GM stimulations. These results also suggest that the ability to identify an injured tendon using this methodology may vary by stimulated muscle, as presence of a tendon injury was not correlated with angular difference during SOL stimulations. Moreover, these results from our low‐loading protocol corroborate prior evidence of altered subtendon function past the 6‐month timepoint post‐rupture thus opening the door for future work applying this protocol to patients at earlier timepoints during injury recovery. The absence of differences between limbs in this outcome measure in healthy adults compared to those with Achilles tendon ruptures suggests that angular difference may be a metric of tendon healing using the uninjured contralateral limb as a reference. While our data demonstrate that the magnitude of angular difference decreases in injured compared to uninjured tendons, the precise physiological meaning of these values in relation to overall muscle–tendon structure and function should be a subject of future investigation.

Interestingly, linear regression analysis revealed that presence of a tendon injury was only significantly correlated with angular difference during GL and GM stimulations. There may be several reasons for this finding. First, the SOL is located deeper than the gastrocnemii and comprises the largest physiological cross‐sectional of the triceps surae complex (63%) compared to the GL (13%) and GM (24%) [33], making it more difficult to isolate during transcutaneous neuromuscular electrical stimulation. It is also possible that placement of surface stimulation electrodes may not optimally activate motor points equally during each muscle stimulation. Motor point locations in the lower limbs demonstrate significant variability among individuals [34], so there is a higher likelihood of electrode placement over a motor unit in the GL and GM given their smaller cross‐sectional areas. Another possible explanation for these results may be injury‐driven changes in select triceps surae muscles. For example, Crouzier and colleagues found that in patients with Achilles tendinopathy, the GL physiological cross‐sectional area was significantly reduced compared to controls and experienced lower activation relative to the GM and SOL muscles during maximal isometric voluntary plantar flexion [35]. The authors speculated that these findings may suggest a relationship between GL contribution to triceps surae force and Achilles tendinopathy symptoms. While such investigation has not yet been conducted on individuals following Achilles tendon rupture, our data show that this tool is more sensitive to changes in subtendon function resulting from gastrocnemius perturbations. Future work should investigate whether these muscle‐specific outcomes are linked to injury‐driven changes in triceps surae function.

Our results also suggest that Achilles tendon rupture injuries alter subtendon function beyond the initial repair site. Imaging position was not significantly correlated with angular difference during any muscle stimulation. These results suggest that acute tendon ruptures reduce functional subtendon independence regardless of the location relative to the initial tear site along the free tendon. A study by Beyer and colleagues also found that relative sliding between superficial and deep layers of the Achilles tendon was significantly reduced in individuals at least 1 year post‐rupture even in regions farthest away from the repair site which were relatively free of scar tissue [6]. These findings suggest that while Achilles subtendon sliding is likely reduced by fibrotic scar tissue at the initial repair site, other mechanisms such as collagen reorganization [8] or altered triceps surae dynamics [36] following rupture may further alter subtendon function. Identifying the origins of these extended functional changes in subtendon behavior would provide insight into improved repair or rehabilitation strategies to mitigate the long‐term structural and functional deficits seen after Achilles tendon injury.

This study has several limitations. First, we examined a relatively small cohort of 19 injured and 17 healthy adults, and the injured cohort received a mix of operative and nonoperative treatments and postoperative instructions from different clinicians. However, literature reports show that outcomes from nonsurgical treatment were acceptable and clinically similar to those from surgical approaches [24]. Additionally, we examined patients at a large range of timepoints postinjury. Though all patients were considered functionally cleared by their physicians to return to physical activity and sport at these timepoints, there is still a potential effect of time postinjury on the degree of subtendon load sharing captured by our methods. The present study is underpowered for such an analysis. Future work may investigate the effects of, and interactions between, time postinjury and treatment type on subtendon function. A post hoc power analysis also revealed that our sample size of 19 patients achieved a power of 0.49 to identify differential angular difference values between injured and uninjured legs (effect size 0.48). A larger sample size of at least 38 patients is required to achieve a power of 0.80. Because angular difference is a novel metric presented in this study, we computed our initial required sample size based on bilateral differences in cluster centroid positions as described in previously published work [25]. Additionally, due to the exploratory nature of is study, it is underpowered for multivariable regression analysis. Consequently, the regression results are intended to elucidate potential predictors of subtendon function since tendon injury is highly multifactorial. Based on the known sex effects on tendon healing [37], we included sex as a variable in our multiple regression analysis. However, due to the discrepancies in rates of Achilles tendon ruptures between males and females [1, 38], these results are likely not reflective of the larger effects of sex on tendon health in the broader population. However, our findings provide critical data to design and power future longitudinal studies aimed at assessing Achilles tendon mechanics throughout tendon healing. Additionally, our injured and uninjured groups were not age‐matched. Aged tendons show reduced subtendon sliding when imaged in the sagittal plane [39] as well as altered interfascicular matrix properties [40, 41], so it is important to investigate changes in our outcome measures across a broader age range of individuals to establish baseline metrics of subtendon load sharing in uninjured tendons. Our regression analysis found that age only increased angular difference values by a small percentage (2.6%), and it is likely that a larger sample size across a broader age range will clarify these effects. We also evaluated the individuals in our injured cohort at only one timepoint, limiting our ability to understand changes in subtendon behavior throughout healing. However, as our prior work has demonstrated that this point tracking method has high intersession reliability [25], future work should apply these methods to individuals at multiple timepoints to assess tendon healing progression. This high intersession reliability coupled with the low tendon loading during our neuromuscular electrical stimulation protocol highlights the potential clinical utility of this imaging paradigm to assess tendon healing during the first 6–12 weeks postinjury when rehabilitation loading has its greatest therapeutic potential.

In conclusion, this study introduced a new potential biomarker of Achilles tendon health to identify altered subtendon function following rupture. Our findings revealed relationships between tendon injury status and the degree of subtendon load sharing 6–12 months postinjury. Our results also show that rupture injuries alter Achilles subtendon function even at locations proximal and distal to the injury site, suggesting that additional factors outside of the initial rupture event may produce lasting changes in subtendon behavior. Future work should investigate changes in subtendon load sharing throughout injury recovery, between different tendon repair strategies, or link subtendon function to patient activity levels. Such work would allow clinicians to make more informed decisions regarding patients' return to sport and daily physical activity and draw broader conclusions between altered subtendon behavior and triceps surae muscle–tendon structure and function.

## Author Contributions

**Kathryn S. Strand:** conceptualization, methodology, formal analysis, investigation, data curation, visualization, writing – original draft. **Todd J. Hullfish:** methodology. **Maggie M. Wagner:** methodology. **Devyn Russo:** investigation. **Max Zawel:** investigation. **Douglas E. Schaubel:** methodology, formal analysis. **Casey Jo Humbyrd:** methodology. **Josh R. Baxter:** conceptualization, methodology, formal analysis, funding acquisition, investigation, writing – original draft. All authors read and critiqued the manuscript extensively and agreed on the final version.

## Conflicts of Interest

The authors declare no conflicts of interest.

## Supporting information


**Figure S1:** Example output from a trial during which the point tracker failed. **Figure S2:** Displacement vectors from each participant in the injured cohort during GL stimulations (images taken at the 3 cm imaging position). **Figure S3:** Displacement vectors from each participant in the uninjured cohort during GL stimulations (images taken at the 3 cm imaging position).
