# Supplementary material for: A Novel Measurement of Altered Achilles Subtendon Load Sharing 6–12 Months Following Rupture
Source: J Orthop Res. 2026 Mar 26;44(4):e70182. doi: 10.1002/jor.70182 (PMC13022063; doi:10.1002/jor.70182)
Supplement: Supplementary file 1 — Figure S1: Example output from a trial during which the point tracker failed. Figure S2: Displacement vectors from each participant in the injured cohort during GL stimulations (images taken at the 3 cm imaging position). Figure S3: Displacement vectors from each participant in the uninjured cohort during GL stimulations (images taken at the 3 cm imaging position). [file JOR-44-0-s001.docx]

**Supplemental Figures**


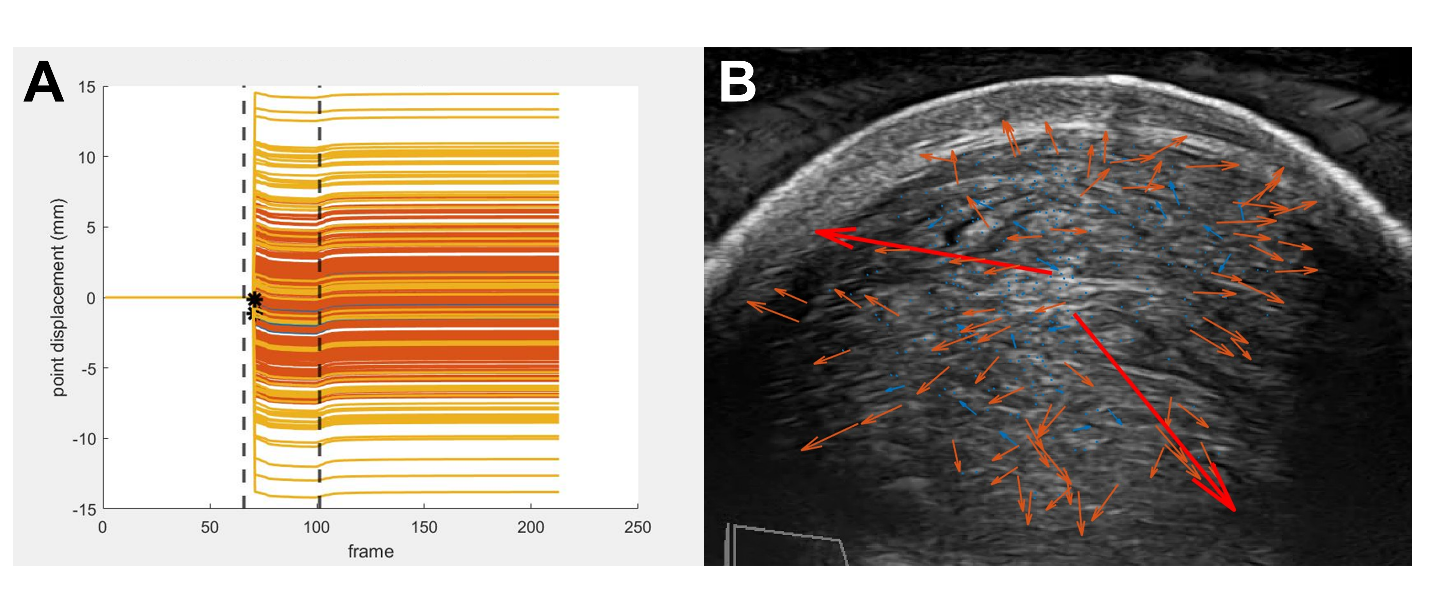


**Figure S1.** Example output from a trial during which the point tracker failed. A) Displacement tracked during trial displays non-physiological levels of tissue motion. B) Displacement vectors for each point plotted over tendon cross section. Large arrows represent the mean direction of vectors in each cluster. Such trials were removed from the final analysis.


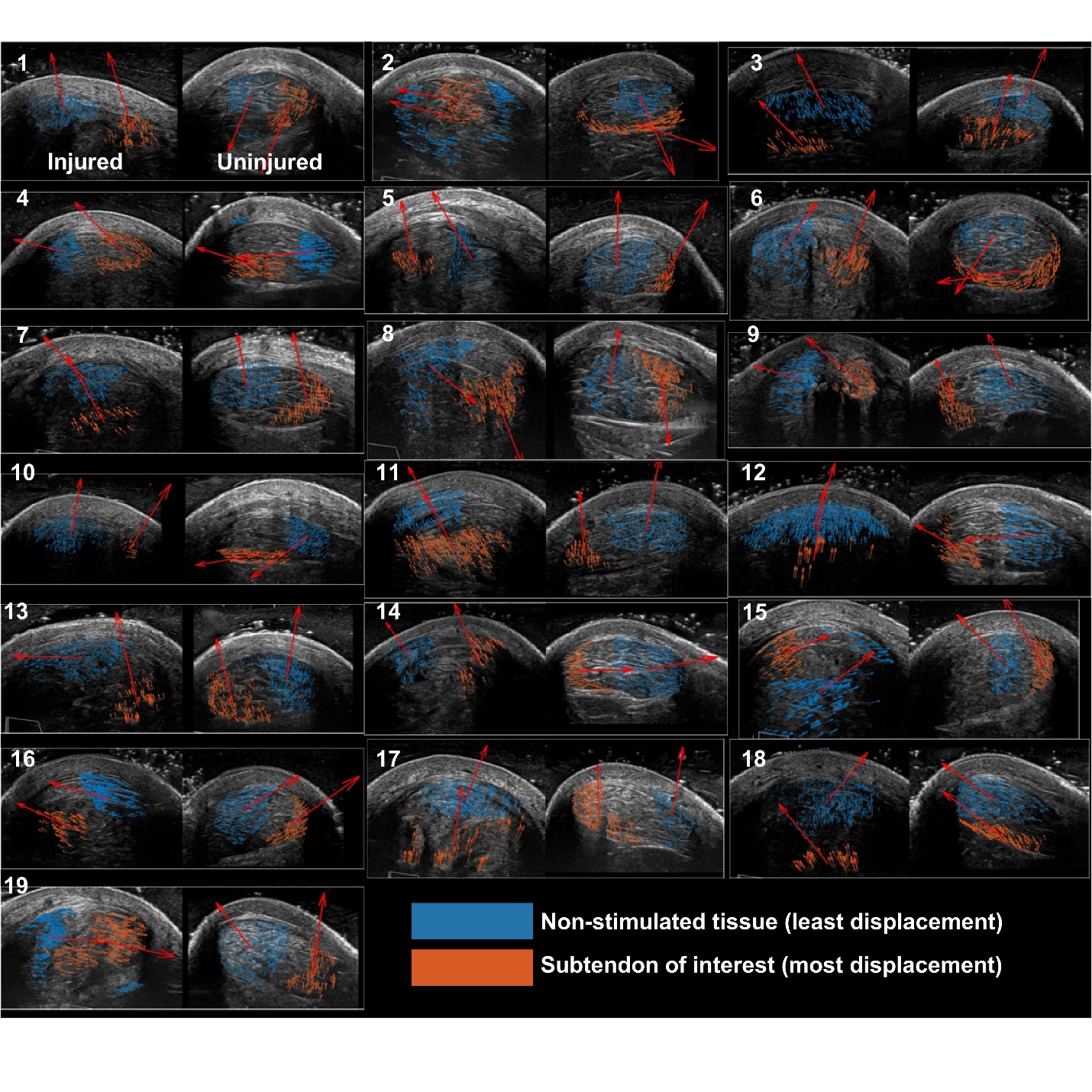


**Figure S2.** Displacement vectors from each participant in the injured cohort during GL stimulations (images taken at the 3 cm imaging position). The large arrow represents the mean displacement direction of all points in each cluster. Each panel displays the injured and uninjured legs from a single subject.


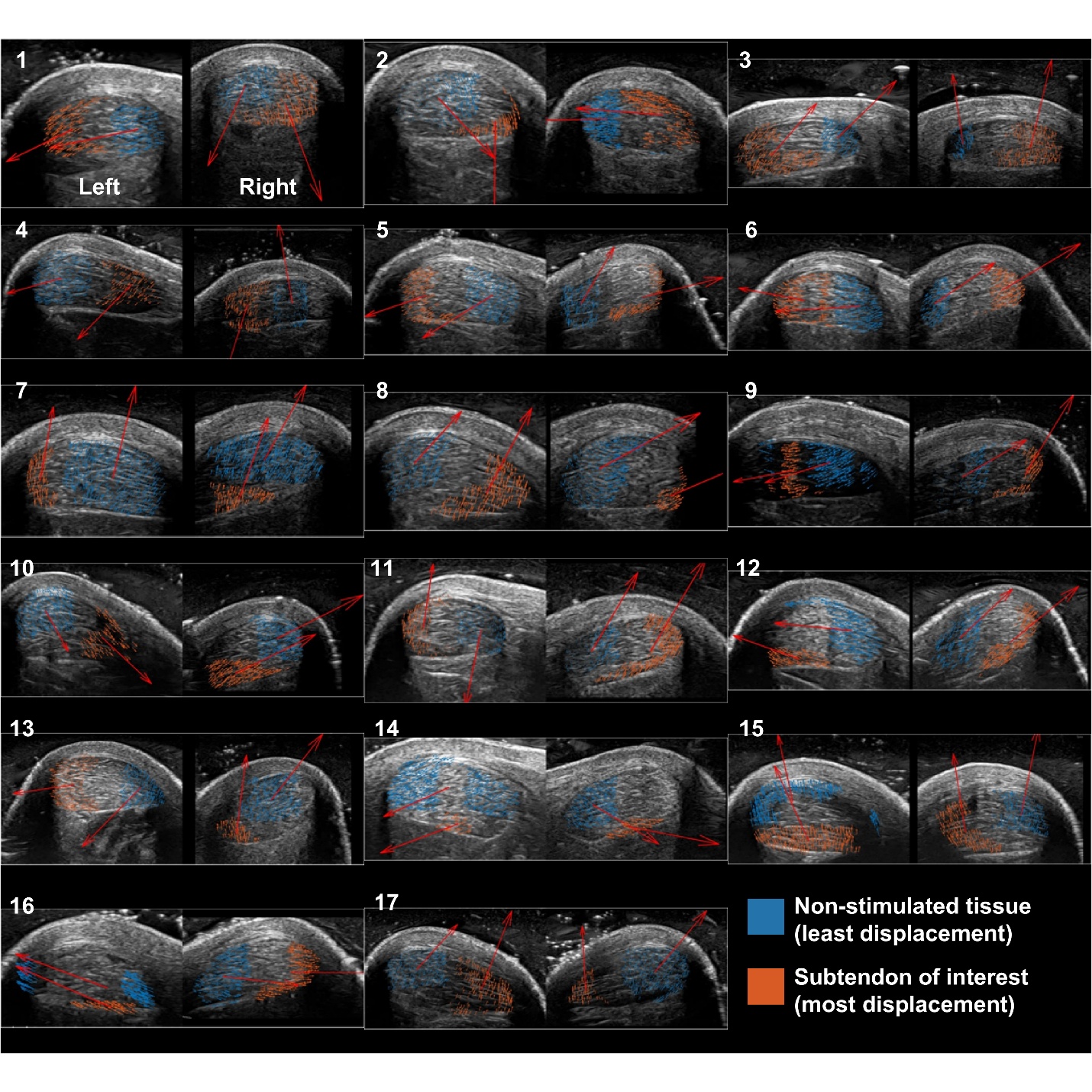


**Figure S3.** Displacement vectors from each participant in the uninjured cohort during GL stimulations (images taken at the 3 cm imaging position). The large arrow represents the mean displacement direction of all points in each cluster. Each panel displays the left and right legs from a single subject.
